# Component orientation measurements in total hip arthroplasty using an inertial measurement unit-based smart trial system

**DOI:** 10.1186/s42836-025-00312-3

**Published:** 2025-06-25

**Authors:** Hao Tang, Yixin Zhou, Baojun Mai, Binjie Zhu, Ping Chen, Yujia Fu, Guangzhi Wang

**Affiliations:** 1 Department of Orthopaedic Surgery, Beijing Jishuitan Hospital, Capital Medical University, National Orthopeadic Center of China, Beijing, 100035 China; 2 Beijing Yiemed Medical Technology Co., Ltd., Beijing, 100098 China; 3 School of Biomedical Engineering, Tsinghua University, Beijing, 100084 China

**Keywords:** Total hip arthroplasty, Anteversion, Inclination, Inertial measurement unit, Smart trial system, Computer-assisted surgery

## Abstract

**Background:**

Intraoperative measurement of component orientation represents a basis for optimizing outcomes after total hip arthroplasty (THA). Although the use of computer navigation systems in THA has improved the accuracy of component positioning, they have not gained widespread popularity due to their complexity, time demands, and time-consuming protocols.

**Methods:**

We developed an Inertial Measurement Unit-based Hip Smart Trial system (IMUHST) to assist with intra-operative monitoring of hip posture. An in vitro validation experiment was conducted using a sawbones with a three-dimensional (3D) measurement model as the reference standard.

**Results:**

The absolute mean error, Bland–Altman analysis, and Intra-class Correlation Coefficient revealed that the accuracy and precision of this system meet the threshold for clinical application.

**Conclusions:**

In conclusion, this in vitro validation demonstrates that the IMUHST system provides accurate component orientation measurements while eliminating the cost and complexity of optical navigation, offering a practical solution for widespread adoption.

Video Abstract

**Supplementary Information:**

The online version contains supplementary material available at 10.1186/s42836-025-00312-3.

## Introduction

Although total hip arthroplasty (THA) has achieved predictable success worldwide, risks remain, including mechanical complications such as dislocation, wear, loosening, and component impingement, which are commonly attributed to component malposition [1–6]. In this regard, acetabular cup orientation and stem anteversion are the two most critical factors for component alignment. The traditional method(s) for intraoperative evaluation of cup and stem orientation rely on the subjective judgment of the surgeon, which has been reported previously to be inaccurate and unreliable [7–9].

The difficulties in determining a reference line or plane are the major reasons for errors in traditional component measurement methods. On the acetabular side, the operative anteversion (OA) and operative inclination (OI) angles assessed intraoperatively by surgeons differ from postoperative radiographic anteversion (RA) and radiographic inclination (RI) [10]. The RA represents the angle between the cup axis and the coronal plane of the body. The RI is defined as the angle between the projection of the cup axis on the coronal plane and the horizontal plane of the body. The anteversion and inclination of the acetabular cup can be defined based on the anatomical Lewinnek plane or functional reference plane, neither directly observable intraoperatively [11, 12]. Moreover, the substantial variation of pelvic positioning also contributes to the inaccuracy of subjective observations [11, 13–15]. On the femoral side, the clinical evaluation of femoral stem anteversion was also reported to be unreliable when compared with navigation [16]. Similarly to the acetabular side, the referenced frame of the stem version (the trans-epicondylar axis or the posterior chondylar line) was estimated subjectively and varied with body posture in traditional assessment [16].

To improve intraoperative accuracy, computer-assisted surgery (CAS) and robot-assisted surgery (RAS) systems for THA have utilized optic navigation as a means of guiding component orientation measurements [17–20]. Computer-assisted navigation accuracy has improved compared to traditional subjective assessments [21, 22]. Despite this, these systems have not yet garnered widespread use, primarily secondary to significant increases in cost. It has been estimated that < 3% of THA surgeries were performed with computer-assisted techniques, owing to multiple barriers such as the high cost of specialized hardware, the time-consuming nature of the process, the invasiveness of fiducial marker placement, and incompatibility with standard surgical workflows [20, 23].

The Inertial Measurement Unit (IMU) is a combination of sensors, including accelerometers, gyroscopes, and magnetometers, and has been widely applied in sports monitoring and surgeries as a means of tracking the spatial orientation of the human body and surgical tools [24–27]. We developed an Inertial Measurement Unit-based Hip Smart Trial system (IMUHST) to accurately monitor intraoperative hip posture and evaluate component orientation in THA, which is independent of complex, expensive optic systems.

**Fig. 1:**
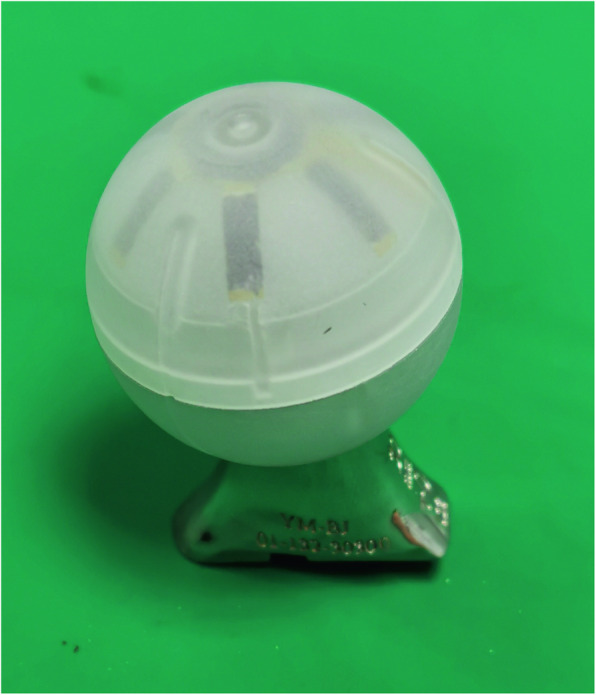
The inertial measurement unit-based hip smart trial system (IMUHST) is composed of a hollow trial head containing IMU sensors mounted onto a trial stem and reduced into the acetabular cup for assessment

The IMUHST system was designed and manufactured by integrating electronic circuits containing IMU sensors into a hollow trial femoral head that is identical to the THA prosthesis utilized (Accolade II, Stryker, USA) (Fig. 1). The spatial orientation of the stem can be dynamically monitored and displayed on the terminal screen in real-time. This provides the system with the non-invasive ability, in comparison with optical systems requiring pin fixation, to accurately monitor the hip joint posture, as well as the potential to measure the orientation of the cup and the stem using specific algorithms.

In this study, an in vitro experiment was conducted to assess the accuracy of intra-operative component orientation measurements taken with the IMUHST system and compared to a THA robot (MAKO, Stryker, Mahwah, NJ, USA) as the reference standard. This study aims to validate the accuracy and reliability of the IMUHST system in measuring acetabular cup orientation and femoral stem anteversion using a robotic 3D reference standard.

## Materials and methods

### Sawbone THA model

**Fig. 2:**
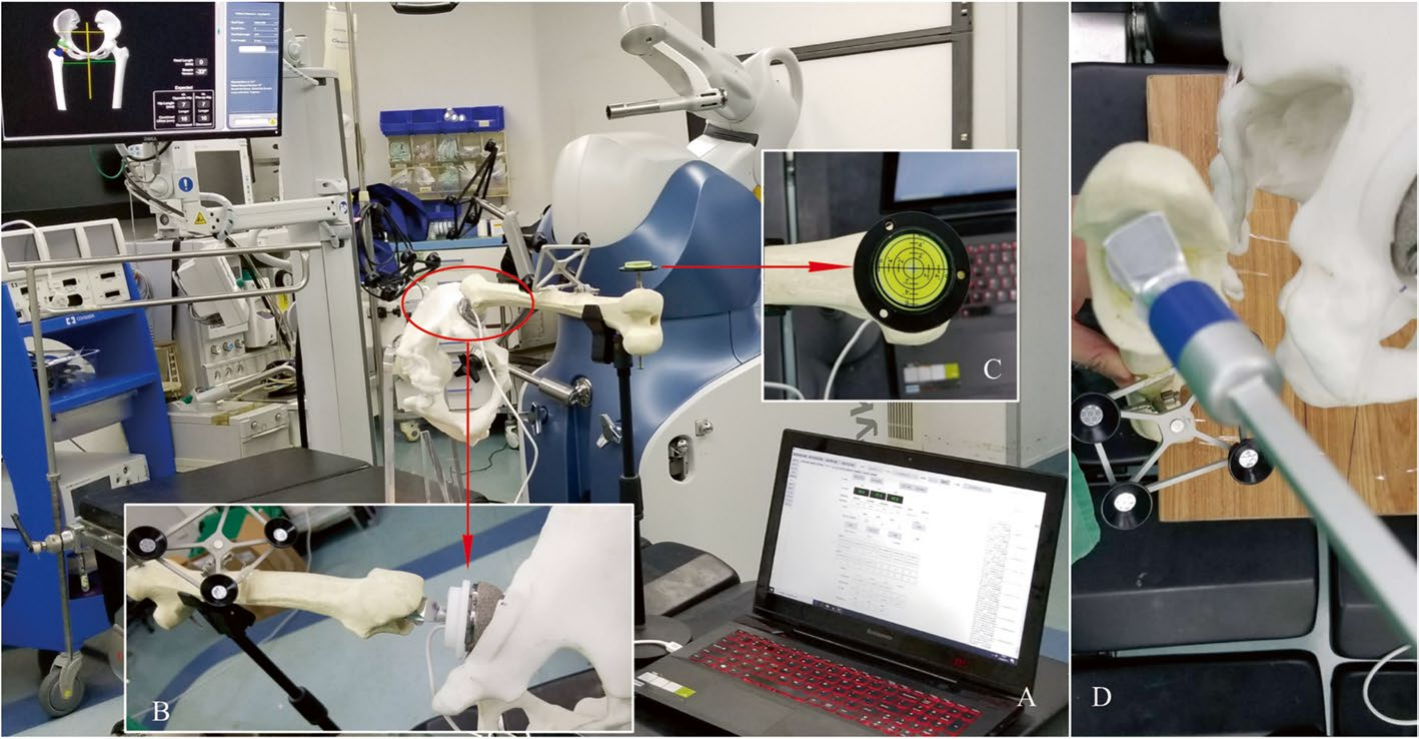
Experimental setting and measurement protocol of the robotic system and IMUHST. **A** The cup is inserted and assessed by the MAKO robotic system as the reference method, and the neutral position of the stem is recorded by the IMUHST to measure the vertical angle between this position and the gravity axis to calculate stem version (SV); **B** The stem neck is then rotated into a position perpendicular to the cup opening plane, guaranteed by a circular plastic mold. The angle between the initial neutral position and the current position is measured as the radiographic anteversion (RA), and the angle between the current stem neck axis and the horizontal plane is measured for calculation of radiographic inclination (RI); **C** The neutral position of the femur is guaranteed by a gradient put on a metal pin passing the trans-epicondyle axis. **D** The stem version can be adjusted for 5° increments between − 50° and 80°, and measured using the robotic system as a reference

Pelvic and femoral models were utilized to simulate the total hip arthroplasty surgery. This in vitro experiment did not use any patient information and thus did not require approval according to the ethics committee of our hospital. We implanted a 56 mm trial cup into the right acetabulum after reaming to the proper size, and a S-ROM femoral stem into the ipsilateral femoral canal after broaching with standard surgical instrumentation (S-ROM, Depuy Orthopaedics, Warsaw, IN, USA). The IMUHST system, incorporated into a 36 mm diameter trial femoral head, was then assembled onto the femoral trial stem and reduced into the cup (Fig. 2).

The pelvis was then fixed onto a plastic scaffold, which was designed to ensure that the anterior pelvic plane was perpendicular to the ground, thereby mimicking the lateral decubitus position utilized in a posterior surgical approach to THA. The whole construct was placed onto a horizontal surgical table surface in the operating theatre. A universal clamp was used to support the weight of the sawbones and to stabilize the posture of the hip to facilitate repeated measurements of stem anteversion.

### Reference system

The MAKO robot THA system (Stryker, Mahwah, NJ, USA) was used to measure the cup RA, RI, and stem anteversion as the reference standard, as it has been validated with a mean absolute error (MAE) of 1°–3° in component orientation measurements [28] (Fig. 2). The acetabular cup was then inserted into the pelvis in various combinations of RA and RI by the MAKO robot system to simulate different orientations of the cup. After insertion, the cup RA and RI values were then double-checked using the validation function of the MAKO system using a probe as the reference for cup orientation. A total of 30 cup positions with RA ranging from 0°–50° and RI ranging from 10°–50° were simulated (Table 1).

**Table 1:** Study design of the measured cup and stem positions

	**Cup RA**	**Cup RI**	**Stem anteversion**
Range	0–50°	10–50°	− 50–85°
Increment	10°	10°	5°
Number of tested angles	6	5	28
Total number of tested positions	30 cup positions		28 stem positions

*RA* Radiographic anteversion, *RI *radiographic inclination

For the femoral side, a redesigned rotatory S-ROM prosthetic model was then inserted to simulate different angles of stem anteversion (Fig. 2). The taper aspect of the stem neck was modified such that it was equivalent to the Accolade II stem (Stryker, Mahwah, NJ, USA) to accommodate accurate mounting of the measurement tool associated with the MAKO robot. The stem was inserted manually into the prepared femoral canal; anteversion was measured by the MAKO system and increased by 5° increments for each position between the range of − 50°–85°. A total of 28 stem positions were simulated (Table 1). The IMUHST system was then utilized to measure stem anteversion for each position, a total of 3 times, including a 30-s interval between measurements.

### Inertial measurement unit-based hip smart trial system

The IMUHST calculates orientation by fusing data from accelerometers, gyroscopes, and magnetometers. This fusion corrects for sensor drift and provides real-time angular measurements relative to gravity and magnetic fields. The IMUHST system presented in this study was composed of measurement hardware in a hollow plastic femoral head and application software in a terminal computer. The trial femoral head and terminal computer were connected via USB cable (Fig. 1). Using the real-time data, the orientation of the smart trial can be read and recorded on the software and then used to measure the orientation of the THA components.

**Fig. 3:**
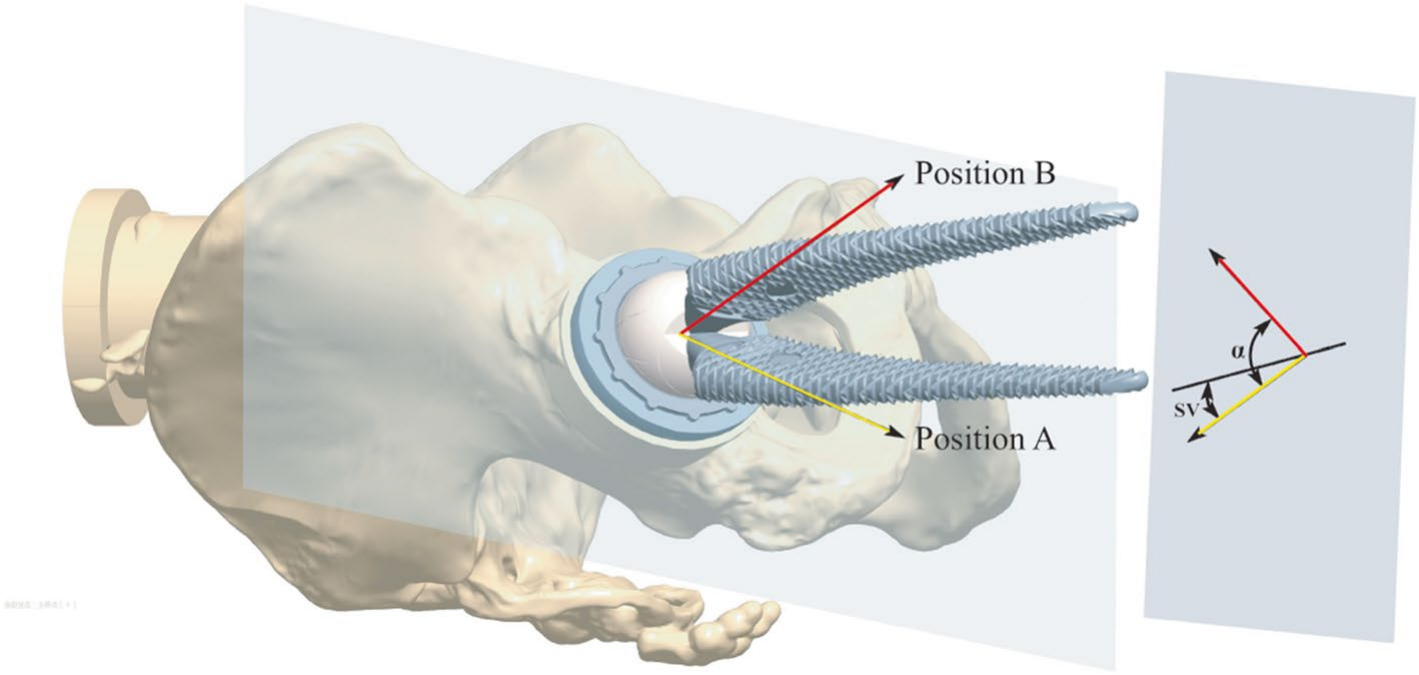
A schematic drawing of the algorithm for radiographic anteversion (RA), radiographic inclination (RI), and stem version (SV) calculations. The femur is first held at a neutral position, with SV measured as the difference between the axial projection of the neck and vertical line; the femur was then flexed and internally rotated to measure RA. RA = f (α − SV)

Stem anteversion was calculated according to the postural change of the IMUHST with respect to a standard neutral position (Fig. 3). With the trans-epicondylar axis along the line of gravity, the projection of the neck central axis onto the axial plane forms the angle of stem version with the vertical line. The method to calculate the RA is listed in the Appendix. The IMUHST and MAKO measurements were each repeated 3 times, respectively, with 10-s intervals between each iteration and for each posture.

### Statistics

With the mechanical posture measurement outcome utilized as the reference standard, we calculated the MAE in component orientation measurement by the IMUHST system with a 95% confidence interval. A Bland–Altman analysis was used to calculate the mean error and the limits of agreement. The Intra-class Correlation Coefficient (ICC) was calculated for each group of postures as an assessment of reliability. All statistical analysis was performed using the SPSS statistical software package (version 15.0; IBM, Armonk, NY, USA) and Medcalc software (Medcalc, Mariakerke, Belgium). The level of significance was set at *P* < 0.05.

## Results

**Fig. 4:**
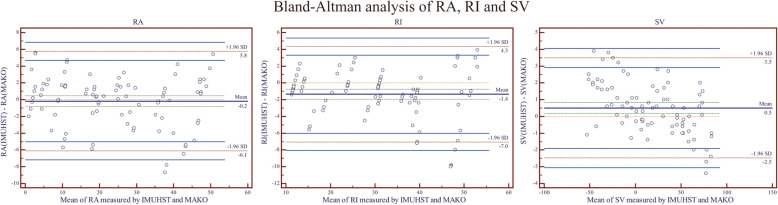
The Bland–Altman analysis of the measured results of the two systems in (**A**). cup radiographic anteversion (RA); **B** cup radiographic inclination (RI); and **C**. stem version (SV) with anteversion marked positive. IMUHST = Inertial Measurement Unit-based Hip Smart Trial system

For the acetabular orientation, the 95% confidence interval of MAE was 2.3 ± 1.9° for measuring radiographic anteversion and 2.2 ± 2.3° for measuring radiographic inclination, respectively. The Bland–Altman analysis data for the cup RA and RI measurements are displayed in Table 2 (Fig. 4).

**Table 2:** Summary for the validity and reliability of the cup and stem orientation measurements

Measurement	MAE (95% CI) (°)	BA Mean Error (°)	BA Limits of Agreement (°)	ICC Value
Radiographic Anteversion (Cup)	2.3 ± 1.9	− 0.2	− 6.1 to 5.8	0.993
Radiographic Inclination (Cup)	2.2 ± 2.3	− 1.4	− 7.0 to 4.3	0.998
Femoral Stem Version	1.2 ± 0.8	0.5	− 2.5 to 3.5	0.994

*MAE* Mean absolute error, *CI *Confidence interval, *BA *Bland–Altman analysis, *ICC *Intra-class Correlation Coefficient

For the femoral stem version, the 95% confidence interval of MAE was 1.2° ± 0.8° by comparison to the robotic measurement. The Bland–Altman analysis showed that the mean of errors was 0.5° in the stem version by comparison to the robotic measurement, and the limitation of the agreement was between − 2.5 to 3.5° in the stem version.

The ICC values of the component orientation measurements by the IMUHST system, repeated 3 times with 30-s intervals, were 0.993, 0.998, and 0.994 for cup radiographic anteversion, cup radiographic inclination, and stem version, respectively.

The static drift measurement revealed that the drift of the current IMUHST ranged between − 0.004 to − 0.001°/s for cup anteversion, < 0.001 to 0.001°/s for cup inclination, and − 0.001 to < 0.001°/s for stem version.

## Discussion

This study evaluated the accuracy and reliability of a novel IMUHST developed for intraoperative component orientation measurements in THA. We previously studied the accuracy and reliability of monitoring hip postures in THA. The orientations of the cup and stem are of critical importance to minimizing complications and improving postoperative hip function and survivorship after THA. Traditional assessment of cup and stem orientation relied on a surgeon’s subjective judgment, which is inherently influenced by a variety of factors, including patient positioning, surgical approach, and point of view, therefore leading to low reliability and validity [30]. By reducing component malposition, the IMUHST may lower dislocation rates (< 2% in navigated THA), enhance reproducibility, and improve long-term implant survivorship of THA.

Computer-assisted THA has been reported to be more accurate in terms of prosthesis positioning than traditional manual methods [20, 31]. The mean absolute error of 1°–2.5° for cup orientation and 1.2° for stem anteversion is clinically acceptable, as deviations exceeding 5° are associated with increased risks of dislocation, impingement, and early implant failure. This is in concert with literature reporting that the average accuracy of IMU-based angle assessment tools reached 2° [32–34].

Although optics-based navigation systems have achieved excellent accuracy for component positioning, leg length, and offset adjustment, it has been reported that navigation is only utilized in approximately 1%–3% of all arthroplasty procedures [20, 23]. This may be due to the increased expense in hardware, complex protocols, and changes to the ordinary workflow of the operative teams, which have impeded widespread adoption. This novel system of IMUHST is advantageous in that it is less invasive as pins for trackers are not required; it is easy to use in terms of incorporating with existing workflow, and it is not dependent on complex and expensive optical navigation hardware [19, 23, 35].

Several IMU-based surgical tools have been invented for hip and knee surgeries. Accelerator sensor systems have achieved promising results in total knee arthroplasty [18, 36, 37]. Silvio et al. developed an IMU-based system specialized for periacetabular osteotomy and reported that the mean difference was < 4 degrees [38]. Recently, a disposable IMU-based navigation system (HipAlign) was developed for THA, which was based on the tracking of instruments by multiple sensors instead of a single trial component. It was reported to achieve an absolute error measurement of 2.6° ± 2.7° (inclination) and 2.8° ± 2.7° (anteversion) by comparison to postoperative CT measurements [39]. The accuracy of measuring anteversion and inclination in our in vitro validation experiments is comparable with those of the HipAlign system, and the limit of agreement in Bland–Altman analysis shows the accuracy is acceptable with ± 10° as the clinically acceptable “safe zone” of deviation from the planned acetabular cup orientation.

Unlike external IMU trackers or optical systems requiring percutaneous pins or separate instruments, the IMUHST embeds sensors into a hollow trial femoral head—a component already used routinely during THA without introducing extra devices. This eliminates additional incisions, reduces invasiveness, and seamlessly integrates into the surgical workflow. To our knowledge, there have been no reports to suggest using an intra-articular IMU-based intraoperative smart trial system to measure component orientation. The creative integration of IMU sensors into the trial femoral head allows accurate and reliable measurements of component orientation with the hip joint reduced. Besides, the IMUHST requires no changes to standard THA instrumentation or surgical steps. The trial head is used identically to conventional trials, avoiding the time-consuming setup of optical trackers or robotic arms. This addresses a major barrier to the adoption of navigation systems.

A potential source of error might emanate from the drift caused by the fluctuations of the local magnetic field. Similar effects of drift on IMU-based monitor systems have been reported previously [32, 40, 41]. All our measurements were conducted in the real environment of an operating theatre. To quantify the drift effect of IMUHST, we repeated the measurement 3 times each within 30 s for each posture, and found that the ICC value ranged from 0.993 to 0.998 for acetabular cup RA, RI, and femoral anteversion measurements. This suggests excellent reliability, indicating minimal static drift over time of the entire device, and is acceptable in a clinical setting. In the current IMUHST system, the static drift was minimized by the 9-axis IMU sensor design, enabling constant minor corrections of drift by monitoring the gravity and magnetic field reference axes, which is more accurate than the traditional 6-axis IMU sensors [42]. While ICC values > 0.99 indicate excellent reliability in vitro, real-world variability (e.g., soft tissue interference) may reduce reproducibility. Ongoing in vivo trials will assess clinical performance.

There are several limitations to this study. First, the in vitro study is unable to fully simulate an intraoperative scenario, as these was devoid of soft tissue and fluid surrounding the sawbones. Second, the IMU sensor accuracy relies on a stable local electromagnetic environment; therefore, strong magnetic field disturbances should be avoided during use. Future iterations will incorporate real-time magnetic field calibration to minimize interference. Third, the current study assumed that the pelvis remained stationary during measurement(s), but for in vivo scenarios, there is coordinated movement between the spine, pelvis, and hip [43]. While pelvic fixation was necessary for controlled experiments, which is unrealistic in clinical settings, future studies will incorporate pelvic trackers containing IMU sensors to monitor the real-time pelvic position.

## Conclusion

The results of the current study revealed that the IMUHST system provides accurate cup orientation and stem version measurements in an in vitro setting, and is a promising sensor system for assessing intraoperative component position(s) in the setting of THA surgery to improve the intraoperative accuracy of component positioning while avoiding the complexity and cost of conventional computer navigation systems. Pending further in vivo validation, the IMUHST holds the potential for commercialization as a portable, workflow-compatible tool for THA.

## Data Availability

The data will be available at request.

## Appendix

The orientation of the acetabular cup was calculated by using the IMUHST as a compass (Fig. 2). In this regard, the Lewinnek plane of the pelvis was used as the reference system [10]. The hip joint was held in a neutral position with the pelvis in an orthogonal posture, which was then recorded with the software. Thereafter, the smart trial was rotated to position the femoral neck perpendicular to the opening plane of the acetabular cup; this was confirmed by the parallelism between the bottom plane of the trial head and the equator of the cup as the second plane (Fig. 2B). The central axes of the neck were projected onto the axial plane of the patient, at which point the difference between the anteversion of the two postures was calculated as the angle “α”, and the anatomical anteversion (AA) of the cup was calculated as AA = α – SV. This was then converted into radiographic anteversion (RA) for comparison with the values in the MAKO robot system using the equation previously published (Fig. 3) [10, 29].
